# Supplementary material for: Tens of thousands additional deaths annually in cities of China between 1.5 °C and 2.0 °C warming
Source: Nat Commun. 2019 Aug 6;10:3376. doi: 10.1038/s41467-019-11283-w (PMC6684802; doi:10.1038/s41467-019-11283-w)
Supplement: Supplementary file 1 — Supplementary Information [file 41467_2019_11283_MOESM1_ESM.pdf]

Supplementary Information

to

Tens of thousands additional deaths annually in cities of China between 1.5ºC and 2.0ºC warming

Wang et al.

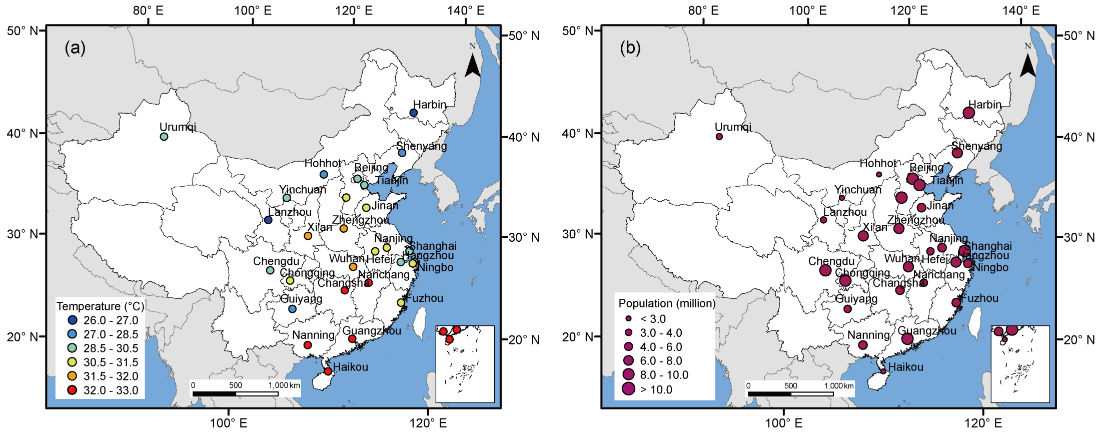

Supplementary Fig. 1 Multi-year averaged summer temperature for the period 1961-2015 (a) and the population in 2010 (b) for 27 major cities in China.

The 27 metropolitan areas of China include 4 municipalities, i.e. Beijing, Tianjin, Shanghai and Chongqing, 22 provincial capitals, i.e. Changsha, Chengdu, Fuzhou, Guangzhou, Guiyang, Harbin, Haikou, Hangzhou, Hefei, Hohhot, Jinan, Lanzhou, Nanchang, Nanjing, Nanning, Shenyang, Shijiazhuang, Wuhan, Urumqi, Xi'an, Yinchuan, Zhengzhou, and 1 metropolitan region in Zhejiang province, Ningbo.

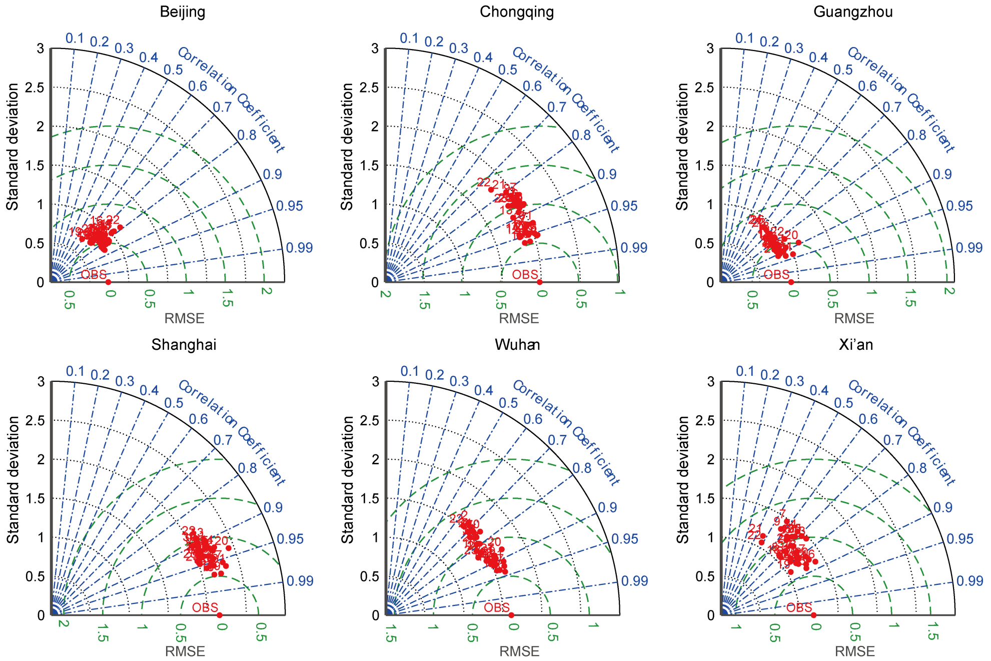

Supplementary Fig. 2 Taylor diagram of high temperature in the major cities of China between GCMs and observation.

Each dot denotes one GCM run, which was bias-corrected by Equidistant Cumulative Distribution Functions (EDCDF) method with refer to observational data, and statistically downscaled to 0.5° resolution by the spatial disaggregation (SD) method [1, 2]. The correlation coefficients between the GCM outputs and the observational field pass the significance test ($p<0.05$), and the standard deviations and RMSEs of two series are within 0.5-1.5 and 1-2.5, respectively.

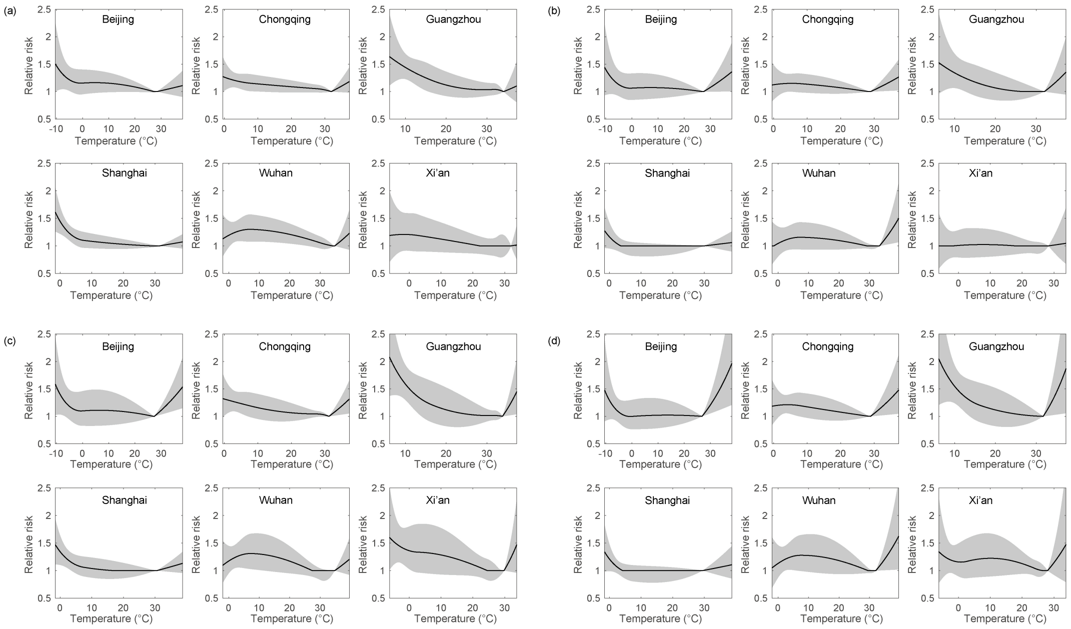

Supplementary Fig. 3 Relative risk of mortality to temperature for male working age population (a), male non-working age population (b), female working age population (c), and female non- working age population (d) for sample cities in China.

The maximum likelihood estimates of RRs are shown as smooth lines and the point-wise 95% confidence intervals are shown as the gray shading

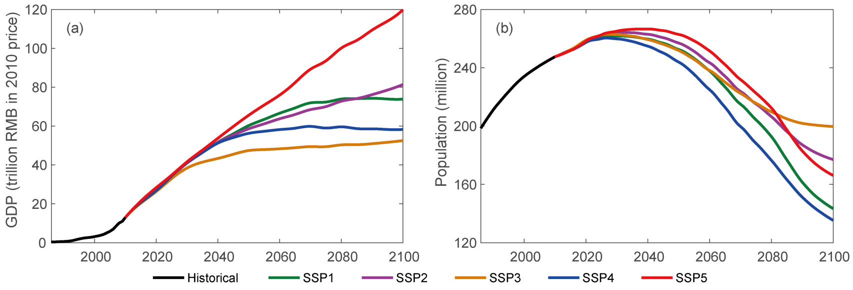

Supplementary Fig. 4 Projected GDP (a) and population (b) under SSPs for the 27 major cities in China.

It is assumed that population will be in low fertility, low mortality, medium migration, and high education for SSP1; in medium fertility, medium mortality, medium migration and medium education for SSP2; in high fertility, high mortality, low migration and low education for SSP3; in high fertility, high mortality, medium migration, and polarized education for SSP4; in low fertility, low mortality, high migration, and high education for SSP5. Annual GDP in the 21st century under the SSPs is obtained with regionalized parameters and newly projected labor force, and standardized to 2010 price to maintain the homogeneity of data series.

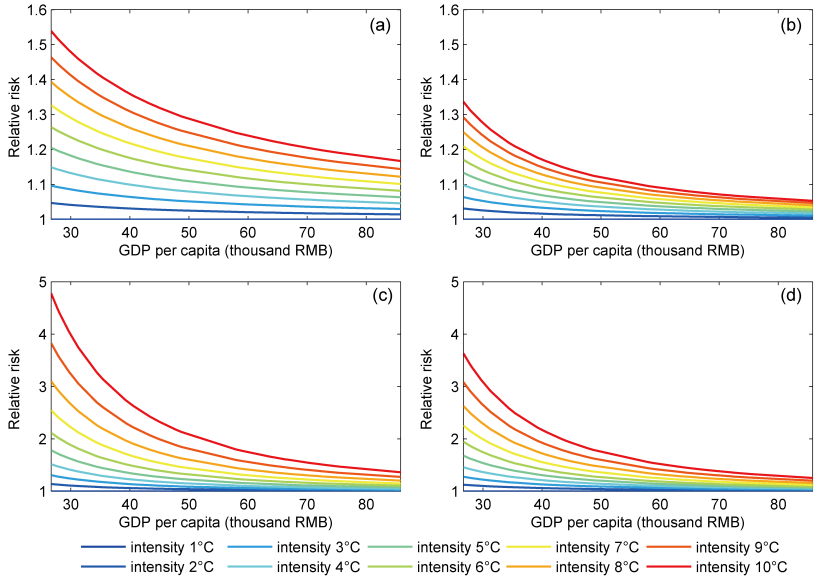

Supplementary Fig. 5 Changes in relative risk of heat-related mortality with the GDP per capita for different gender and age groups: male working age population(a); male non-working age population(b); female working age population(c); and female non-working age population(d).

The GDP per capita in metropolises and their relative risk to high temperature with different intensities are fitted according to equation

$$RR_I = \frac{a_I}{\left({}^{\mathrm{GDP}_S}/{}_{\mathrm{POP}_S}\right)^2} + \frac{b_I}{\left({}^{\mathrm{GDP}_S}/{}_{\mathrm{POP}_S}\right)} + 1$$

Here, $RR_I = \{RR_{I,s=1},\ RR_{I,s=2}, RR_{I,s=3}, \ldots RR_{I,s=27}\}$; parameters a_I and b_I can be fitted by the least-square method according to the relative risk estimated $(RR_{I,s})$ by the DLNM; GDP and POP are from records of the observational period; s represents the different cities; I represents intensity of high temperature. Accordingly, improved adaptation capacity (AC_I) can be quantified by calculating the percentage changes of $RR_{I,s}$ under different socioeconomic conditions relative to reference period. The colored lines denote the intensities of high-temperature.

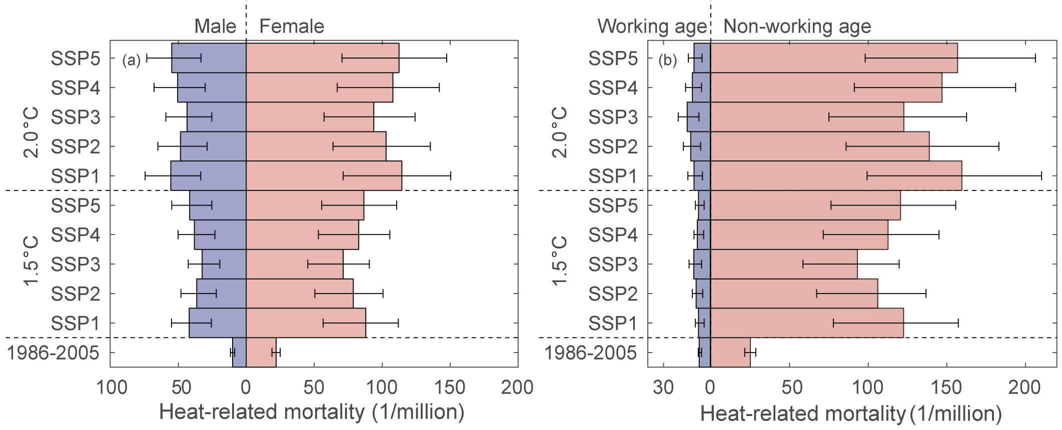

Supplementary Fig. 6 Annual gender (a) and age(b) specific heat-related mortality at the reference period (1986-2005), 1.5ºC and 2.0ºC global warming under SSPs without consideration of improved adaptation capacity.

Colored bars and black straight lines denote the ensemble mean and range of mortality estimated by multiple GCMs.

Supplementary Table 1 The multi-year averaged summer temperature in 1961-2015, the population in 2010, and the annual mortality averaged for 2007-2013 in 27 China major cities.

Name	Averaged summer temperature (ºC)	Population size (million)	Total annual mortality
Beijing	30.5	19.6	69,936.0
Changsha	32.3	7.0	10,221.8
Chengdu	28.6	14.0	79,874.0
Chongqing	30.7	28.8	168,761.1
Fuzhou	31.4	7.1	19,613.4
Guangzhou	32.4	12.7	41,703.8
Guiyang	27.5	4.3	26,587.4
Harbin	26.3	10.6	42,634.1
Haikou	32.8	2.0	7,193.0
Hangzhou	28.5	8.7	24,112.1
Hefei	31.3	5.7	8,433.8
Hohhot	27.5	2.9	11,672.5
Jinan	31.4	6.8	29,919.2
Lanzhou	26.6	3.6	9,798.0
Nanchang	32.2	5.0	7,616.2
Nanjing	31.1	8.0	22,919.8
Nanning	32.6	6.7	25,371.2
Ningbo	31.3	7.6	20,998.2
Shanghai	30.4	23.0	56,834.4
Shenyang	28.2	8.1	33,085.4
Shijiazhuang	31.5	10.2	44,192.8
Tianjin	29.8	12.9	47,506.4
Wuhan	31.9	9.8	9,022.8
Urumqi	30.1	3.1	13,185.9
Xi’an	31.7	8.5	22,006.6
Yinchuan	28.7	2.0	5,621.8
Zhengzhou	31.6	8.6	13,080.1

Supplementary Table 2 31 outputs from 15 GCMs with different numbers of runs in the CMIP5.

Institute name	Institute ID	Model name	Ensemble runs
Centre National de Recherches Météorologiques/ Centre Européen de Recherche et Formation Avancées en Calcul Scientifique	CNRM-CERFACS	CNRM-CM5	r1i1p1
Canadian Centre for Climate Modelling and Analysis	CCCMA	CanESM2	r1i1p1, r2i1p1, r3i1p1, r4i1p1, r5i1p1
Commonwealth Scientific and Industrial Research Organization/Queensland Climate Change Centre of Excellence	CSIRO-QCCCE	CSIRO-Mk3.6.0	r2i1p1, r3i1p1, r4i1p1, r5i1p1, r6i1p1, r7i1p1, r8i1p1, r9i1p1, r10i1p1
Geophysical Fluid Dynamics Laboratory	NOAA GFDL	GFDL-CM3	r1i1p1
		GFDL-ESM2G	r1i1p1
		GFDL-ESM2M	r1i1p1
Met Office Hadley Centre	MOHC	HadGEM2-ES	r1i1p1
Institute Pierre-Simon Laplace	IPSL	IPSL-CM5A-LR	r1i1p1
Japan Agency for Marine-Earth Science and Technology, Atmosphere and Ocean Research Institute (The University of Tokyo), and National Institute for Environmental Studies	MIROC	MIROC-ESM	r1i1p1
		MIROC-ESM-CHEM	r1i1p1
		MIROC5	r1i1p1, r2i1p1, r3i1p1
Meteorological Research Institute	MRI	MRI-CGCM3	r1i1p1
Max Planck Institute for Meteorology	MPI-M	MPI-ESM-LR	r1i1p1, r2i1p1, r3i1p1
		MPI-ESM-MR	r1i1p1,
Norwegian Climate Centre, Norway	NCC	NorESM1-M	r1i1p1

Supplementary Table 3 Threshold temperature of heat-related mortality for different gender and age groups in major cities of China. The threshold temperature describes the temperature at which harmful effects start to occur to the population.

Name	Latitude (°N)	Threshold temperature (°C)			
		male working age	male non-working age	female working age	female non-working age
Beijing	39.9	28.4	27.4	27.4	26.8
Changsha	28.2	35.5	33.4	33.2	32.3
Chengdu	30.7	24.8	24.6	24.4	21.1
Chongqing	29.6	31.9	28.9	31.2	28.9
Fuzhou	26.1	33.3	32.8	33.1	32.7
Guangzhou	23.2	34.1	32.0	33.8	31.7
Guiyang	26.6	27.1	26.1	26.7	25.6
Harbin	45.8	29.1	27.5	28.1	26.9
Haikou	20.0	34.9	34.4	34.8	33.5
Hangzhou	30.3	32.2	30.8	31.3	30.1
Hefei	31.9	33.6	30.8	31.6	30.6
Hohhot	40.8	28.2	25.9	25.2	24.7
Jinan	36.7	31.9	30.1	31.1	29.6
Lanzhou	36.0	27.0	24.0	26.0	23.3
Nanchang	28.7	34.8	32.5	34.4	32.3
Nanjing	32.0	30.6	29.4	30.0	28.6
Nanning	22.8	33.9	33.0	33.8	32.5
Ningbo	29.9	31.6	30.1	30.7	29.6
Shanghai	31.2	31.4	30.0	30.6	29.5
Shenyang	41.8	28.6	28.2	28.4	27.8
Shijiazhuang	38.0	29.9	28.8	29.3	28.8
Tianjin	39.1	30.3	29.1	29.8	28.7
Wuhan	43.8	34.8	33.3	34.8	32.1
Urumqi	30.5	28.0	24.7	25.0	24.1
Xi’an	34.3	32.0	28.3	29.8	28.0
Yinchuan	38.5	30.9	30.6	30.9	30.6
Zhengzhou	34.8	33.8	32.5	33.1	31.8

Supplementary Table 4 Annual population and GDP summed over 27 major cities in China

	Reference period	2060-2099 SSP1	SSP2	SSP3	SSP4	SSP5
Total Population (million)	223.1	191.0	207.6	213.5	177.9	210.0
Total GDP (trillion RMB)	2.2	72.5	72.1	50.1	58.9	98.3

Note: GDP is standardized to 2010 price.

Supplementary Table 5 Mortality per million for different gender and age groups in China major cities in 1986-2005 and at 1.5°C and 2.0°C global warming under the assumption that the socioeconomy remains stable at the 1986-2005 average status.

	1986-2005	1.5°C	2.0°C
Total	32.1 (27.6-36.5)	64.3 (41.1-82.2)	85.5 (51.0-113.5)
Male	10.1 (8.6-11.6)	21.0 (12.9-27.6)	28.4 (16.3-38.5)
Female	22.0 (19.0-25.0)	43.3 (28.2-54.6)	57.1 (34.6-74.9)
Working age	7.0 (5.9-8.0)	14.8 (8.9-19.2)	20.2 (10.9-27.9)
Non-working age	25.1 (21.6-28.5)	49.5 (32.1-63.0)	65.3 (40.0-85.5)

Note: numbers in the bracket are estimated range of GCMs

Supplementary Table 6 Mortality per million for different gender and age groups in China major cities in 1986-2005 and at 1.5°C and 2.0°C global warming under five SSPs

	1986-2005	1.5°C					2.0°C				
		SSP1	SSP2	SSP3	SSP4	SSP5	SSP1	SSP2	SSP3	SSP4	SSP5
Total	32.1<br>(27.6-36.5)	65.6<br>(47.3-81.4)	56.8<br>(41.1-70.7)	48.8<br>(35-60.8)	60.5<br>(43.7-75.4)	67.2<br>(48-83.9)	79.0<br>(55.2-95.4)	68.5<br>(47.7-82.6)	59.2<br>(40.4-71.1)	73.0<br>(50.8-88.4)	81.3<br>(56.5-98.5)
Male	10.1<br>(8.6-11.6)	25.5<br>(16.9-33)	21.7<br>(14.3-28.2)	18.5<br>(11.9-24.2)	23.1<br>(15.3-29.9)	26.3<br>(17.3-34)	32.2<br>(20.8-41.2)	27.5<br>(17.7-35.2)	23.7<br>(14.8-30.5)	29.3<br>(18.8-37.4)	33.3<br>(21.5-42.5)
Female	22.0<br>(19.0-25.0)	40.1<br>(30.5-48.5)	35.1<br>(26.8-42.6)	30.3<br>(23-36.6)	37.4<br>(28.5-45.5)	40.9<br>(30.7-49.9)	46.8<br>(34.4-54.7)	41.0<br>(30-47.9)	35.5<br>(25.6-41.1)	43.7<br>(32-51.3)	48.0<br>(35-56.4)
Working age	7.0<br>(5.9-8.0)	2.8<br>(2.4-3.3)	3.4<br>(3.0-4.1)	4.1<br>(3.5-4.9)	3.0<br>(2.6-3.7)	2.8<br>(2.3-3.3)	3.0<br>(2.5-3.4)	3.7<br>(3.1-4.2)	4.5<br>(3.7-5.1)	3.3<br>(2.7-3.7)	3.1<br>(2.4-3.5)
Non-working age	25.1<br>(21.6-28.5)	62.8<br>(44.9-78.4)	53.4<br>(38.1-66.9)	44.7<br>(31.5-56.2)	57.5<br>(41.1-72)	64.4<br>(45.7-80.7)	76<br>(52.7-92.2)	64.8<br>(44.5-78.6)	54.7<br>(36.6-66.6)	69.7<br>(48.1-84.8)	78.2<br>(54.1-95.3)

Note: numbers in the bracket are estimated range of GCMs

Supplementary References

1. Su B, *et al.* Drought losses in China might double between the 1.5 °C and 2.0 °C warming. *Proceedings of the National Academy of Sciences*, **115**, 10600-10605 (2018).
2. Li H, Sheffield J, Wood EF. Bias correction of monthly precipitation and temperature fields from Intergovernmental Panel on Climate Change AR4 models using equidistant quantile matching. *Journal of Geophysical Research: Atmospheres* **115**, D10101 (2010).
